# Microbiome Toolbox: methodological approaches to derive and visualize microbiome trajectories

**DOI:** 10.1093/bioinformatics/btac781

**Published:** 2022-12-05

**Authors:** Jelena Banjac, Norbert Sprenger, Shaillay Kumar Dogra

**Affiliations:** Data Science, Swiss Federal Institute of Technology Lausanne (EPFL), Lausanne 1015, Switzerland; Department of Gastrointestinal Health, Nestlé Institute of Health Sciences, Nestlé Research, Société des Produits Nestlé S.A, Lausanne 1000, Switzerland; Department of Gastrointestinal Health, Nestlé Institute of Health Sciences, Nestlé Research, Société des Produits Nestlé S.A, Lausanne 1000, Switzerland

## Abstract

**Motivation:**

The gut microbiome changes rapidly under the influence of different factors such as age, dietary changes or medications to name just a few. To analyze and understand such changes, we present a Microbiome Toolbox. We implemented several methods for analysis and exploration to provide interactive visualizations for easy comprehension and reporting of longitudinal microbiome data.

**Results:**

Based on the abundance of microbiome features such as taxa as well as functional capacity modules, and with the corresponding metadata per sample, the Microbiome Toolbox includes methods for (i) data analysis and exploration, (ii) data preparation including dataset-specific preprocessing and transformation, (iii) best feature selection for log-ratio denominators, (iv) two-group analysis, (v) microbiome trajectory prediction with feature importance over time, (vi) spline and linear regression statistical analysis for testing universality across different groups and differentiation of two trajectories, (vii) longitudinal anomaly detection on the microbiome trajectory and (viii) simulated intervention to return anomaly back to a reference trajectory.

**Availability and implementation:**

The software tools are open source and implemented in Python. For developers interested in additional functionality of the Microbiome Toolbox, it is modular allowing for further extension with custom methods and analysis. The code, python package and the link to the interactive dashboard of Microbiome Toolbox are available on GitHub https://github.com/JelenaBanjac/microbiome-toolbox

**Supplementary information:**

Supplementary data are available at *Bioinformatics* online.

## 1 Introduction

Microbiome as a concept usually refers to the composition and function of myriads of bacteria in an ecosystem, such as the gut or other body sites of humans or animals (Dogra *et al.*, 2020). The gut microbiome is particularly dynamic during early life development yet is still susceptible to change and malleable and it reaches a stable state at around 2–3 years of age (Cher and Yassour, 2020). Diet is a major factor affecting the microbiome throughout life. For example, the adult microbiome was shown to change in response to drastic changes in diet such as a high-fat or ketogenic diet (David *et al.*, 2014; Mardinoglu *et al.*, 2018). Not surprisingly, as a response to antibiotics, the gut microbiome is also drastically impacted but recovers subsequently to a large extent (Palleja *et al.*, 2018).

Exploring and understanding changes in the microbiome in relation to different factors such as time or age, changes in the environment and diet, as well as medications, are of great interest especially as these relate to health conditions. In early life, an age-appropriate microbiome is understood to be critically important for appropriate immune competence development (Dogra *et al.*, 2015). Equally, numerous examples relate adult gut microbiome features to health conditions (Dogra *et al.*, 2020). Hence, the importance of a Microbiome Toolbox that allows the identification of the key microbiome features that characterize an appropriate microbiome versus one that deviates.

**Fig. 1.**
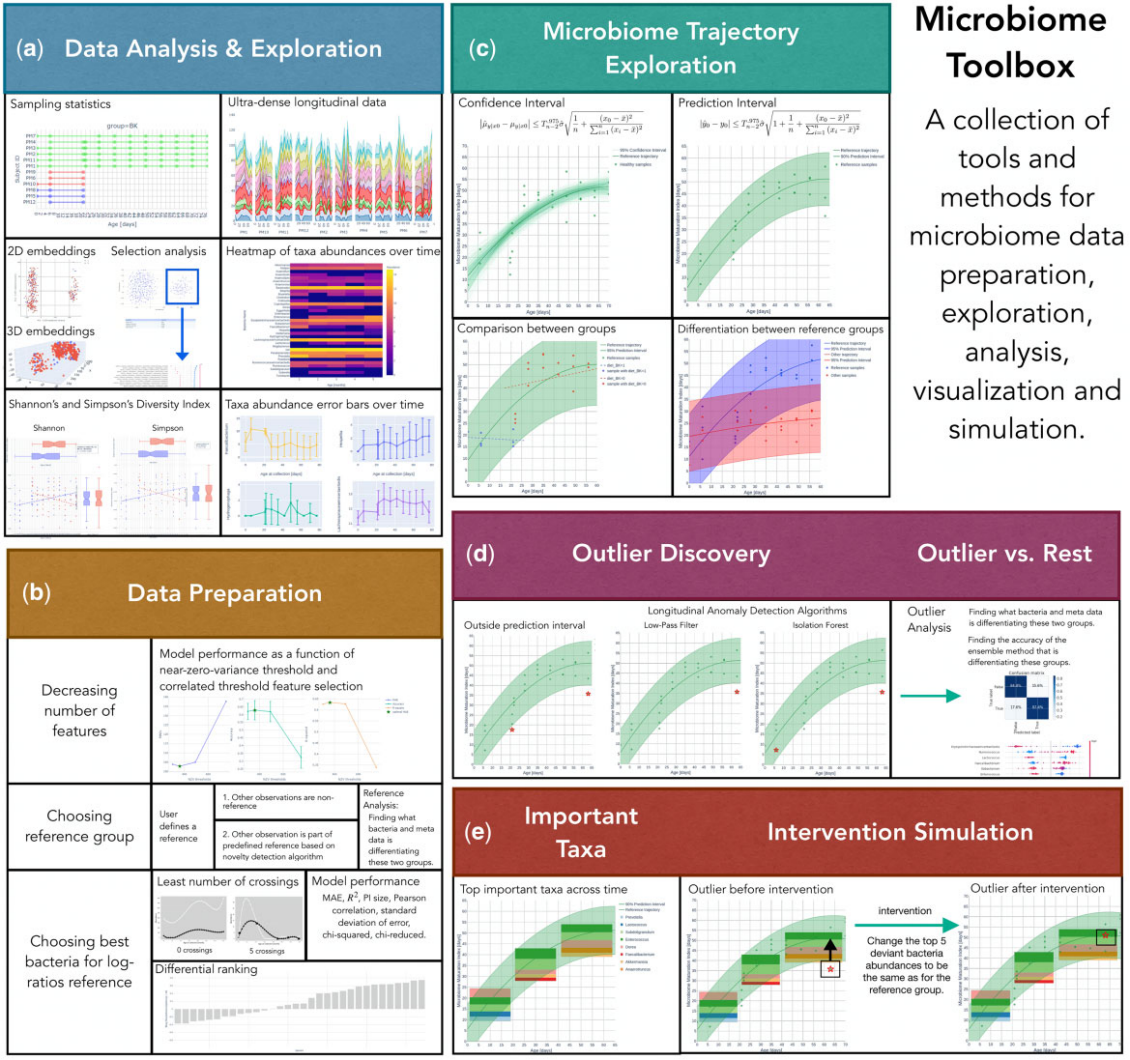
Microbiome Toolbox has multiple components for (**a**) exploration of microbiome data, (**b**) preparing data for subsequent analyses, (**c**) constructing microbiome trajectories, (**d**) determining who is an outlier or within a trajectory and (**e**) identifying important features as possibilities to return an outlier sample back on to the trajectory. The data used to illustrate the functionalities of this Microbiome Toolbox is from the R-package—metagenomeSeq (Paulson *et al.*, 2013). Briefly, 12 germ-free mice fed a low-fat, plant polysaccharide-rich diet, were inoculated with adult human fecal material by gavage. Mice remained on the same diet for 4 weeks before a subset of six mice were switched to a high-fat, high-sugar diet for an additional 8 weeks

Here, we present a Microbiome Toolbox to facilitate such explorations and understanding that can be employed as is for efficient dataset analysis or customized for further data exploitation. Beyond the customary data visualizations and explorations, we implement some specific methods for exploring the microbiome trajectories. Besides working with relative abundances, a key aspect of the calculations is using log ratios to integrate relevant algorithms. We take a machine-learning-based approach to derive a microbiome trajectory. We define on- and off- the trajectory based on various criteria, and we identify key determining features that place a sample on- or off- the trajectory. Furthermore, we suggest what changes can bring a sample back onto the trajectory. An overview of the Microbiome Toolbox is presented in Figure 1.

## 2 Materials and methods

### 2.1 Data analysis and exploration

To get an overview and understanding of the data, Microbiome Toolbox has the plot to visualize the sampling statistics (Fig. 1a). The example data used here, briefly described in the legend of Figure 1, is from the R-package—metagenomeSeq (Paulson *et al.*, 2013), and its source is pointed to in Supplementary Table S1 under ‘mouseData’. We can visualize the microbiome data in an ultra-dense manner. Feature abundances, such as taxa, can be visualized over time as a heatmap or as a median or mean with error bars. Diversity indices such as Shannon or Simpson can be calculated. Embedding plots can be used to visualize the multi-dimensional data in a low-dimensional latent space. Outlier clusters can be identified and further analyzed to identify discriminating features distinguishing these outliers from the rest of the group.

### 2.2 Data preparation

Besides having feature abundances, one can perform a log-ratio transformation (Fig. 1b). For the purpose of calculating this log-ratio, we can identify a suitable bacterium to go in the denominator by using methods such as the least number of crossings, bacteria with very low or very high differential rankings, or choosing the bacteria with the best model performance when used as a denominator. We can also make a ‘hybrid model’ by applying the domain knowledge to incorporate features in addition to the ones identified as important by the model. Next, we can define a reference or control group to be used as a basis for setting the trajectory. We can then classify all other samples into a non-reference group or use an unsupervised novelty detection algorithm to decide whether the observed sample belongs to the reference. Before deriving microbiome trajectories, we first need to prepare the data by removing correlated or low-variance features. We can also choose the subset of taxa the model will be trained on and still achieve an optimal performance.

### 2.3 Microbiome trajectory

Using machine-learning algorithms, we predict a Microbiome Maturation Index (MMI) as a function of time (Fig. 1c). A smooth fit is then used to obtain the trajectory. To interpret the predictions of the model we use SHapley Additive exPlanation (SHAP) values analysis (Lundberg *et al.*, 2020). A confidence interval or prediction interval can be utilized to check the nature of the spread of points on the trajectory. Comparison between groups or references can be made by fitting lines specific to each group and then running statistical tests to determine if these are significantly different. Additionally, we can evaluate trajectory performance using more than 40 different models including a custom-made deep learning model for the dataset—also, different models can be chosen individually for different datasets.

### 2.4 Outlier discovery

Outliers can be identified by various methods such as being outside the prediction interval or by longitudinal anomaly detection algorithms such as a low-pass filter or Isolation Forest (Liu et al. 2008) implemented using a rolling average window. We can then use a machine-learning ensemble model, discriminating between the outliers and the (subsampled) rest of the dataset, to classify these outliers. We can interpret the predictions using SHAP analysis to identify the bacteria or metadata factors that differentiate the outliers from the (subsampled) rest.

### 2.5 Important features and intervention simulation

Lastly, we can identify the key bacteria that are important per the user-defined time window in the trajectory (Fig. 1e). For outliers, some of these key features are out of the normal range causing the deviation from the trajectory. In a simulation setting, by restoring these numbers to be the same as for the key features in the reference group, we can make the outliers move closer to the trajectory. Additionally, we can see what features are shared between the detected outlier samples and compare whether they can be differentiated from the non-outlier samples. This then provides insights into how external factors affect our samples and what could be possible key interventions in real life, such as nutrition-based inteventions, to reposition these outliers back to normal.

## 3 Conclusions

We present here a Microbiome Toolbox to depict microbiome change as trajectories under different conditions such as time, diet changes or perturbations. While the Microbiome Toolbox has implemented intricate methods and complex algorithms, it also provides a rich variety of plots for easy visual comprehension and reporting. We hope that microbiome researchers will find this Microbiome Toolbox particularly useful for the examination of their data and for deriving meaningful insights.

## Supplementary Material

btac781_Supplementary_Data

## References

[btac781-B1] Cher A. , Yassour M. (2020) Chapter 8 - The compositional development of the microbiome in early life. The Human Microbiome in Early Life: Implications to Health and Disease., 177–195.

[btac781-B2] David L.A. et al (2014) Diet rapidly and reproducibly alters the human gut microbiome. Nature, 505, 559–563. 24336217 10.1038/nature12820 PMC3957428

[btac781-B3] Dogra S.K. et al (2020) Gut microbiota resilience: definition, link to health and strategies for intervention. Front. Microbiol., 11, 572921. 33042082 10.3389/fmicb.2020.572921 PMC7522446

[btac781-B4] Dogra S. et al (2015) Rate of establishing the gut microbiota in infancy has consequences for future health. Gut Microbes, 6, 321–325. 26516657 10.1080/19490976.2015.1078051 PMC4826121

[btac781-B5] Liu F. et al (2008) Isolation forest. In: *Eighth IEEE International Conference on Data Mining, ICDM’08, Pisa, Italy.*

[btac781-B6] Lundberg S.M. et al (2020) From local explanations to global understanding with explainable AI for trees. Nat. Mach. Intell., 2, 56–67. 32607472 10.1038/s42256-019-0138-9 PMC7326367

[btac781-B7] Mardinoglu A. et al (2018) An integrated understanding of the rapid metabolic benefits of a Carbohydrate-Restricted diet on hepatic steatosis in humans. Cell Metab., 27, 559–571.e5. 29456073 10.1016/j.cmet.2018.01.005 PMC6706084

[btac781-B8] Palleja A. et al (2018) Recovery of gut microbiota of healthy adults following antibiotic exposure. Nat. Microbiol., 3, 1255–1265. 30349083 10.1038/s41564-018-0257-9

[btac781-B9] Paulson J.N. et al (2013) Differential abundance analysis for microbial marker-gene surveys. Nat. Methods, 10, 1200–1202. 24076764 10.1038/nmeth.2658 PMC4010126

