# Supplementary material for: Microbiome Toolbox: methodological approaches to derive and visualize microbiome trajectories
Source: Bioinformatics. 2022 Dec 5;39(1):btac781. doi: 10.1093/bioinformatics/btac781 (PMC9825749; doi:10.1093/bioinformatics/btac781)
Supplement: btac781_Supplementary_Data [file btac781_supplementary_data.zip › ELM trajectory methods-microbiome-toolbox-revised-R3-Supplementary Information-accepted.pdf]

[SUPPLEMENTARY INFORMATION]

Microbiome Toolbox: Methodological approaches to derive and visualize microbiome trajectories

Jelena Banjac[1], Norbert Sprenger[2] and Shaillay Kumar Dogra[2, *]

[1]Data Science, Swiss Federal Institute of Technology Lausanne (EPFL), [2]Nestlé Institute of Health Sciences, Nestlé Research, Société des Produits Nestlé S.A., Lausanne, Switzerland.

*To whom correspondence should be addressed

Contact: ShaillayKumar.Dogra@rd.nestle.com

Interactive Dashboard

A web-based interactive dashboard is available at:

https://microbiome-toolbox.azurewebsites.net/

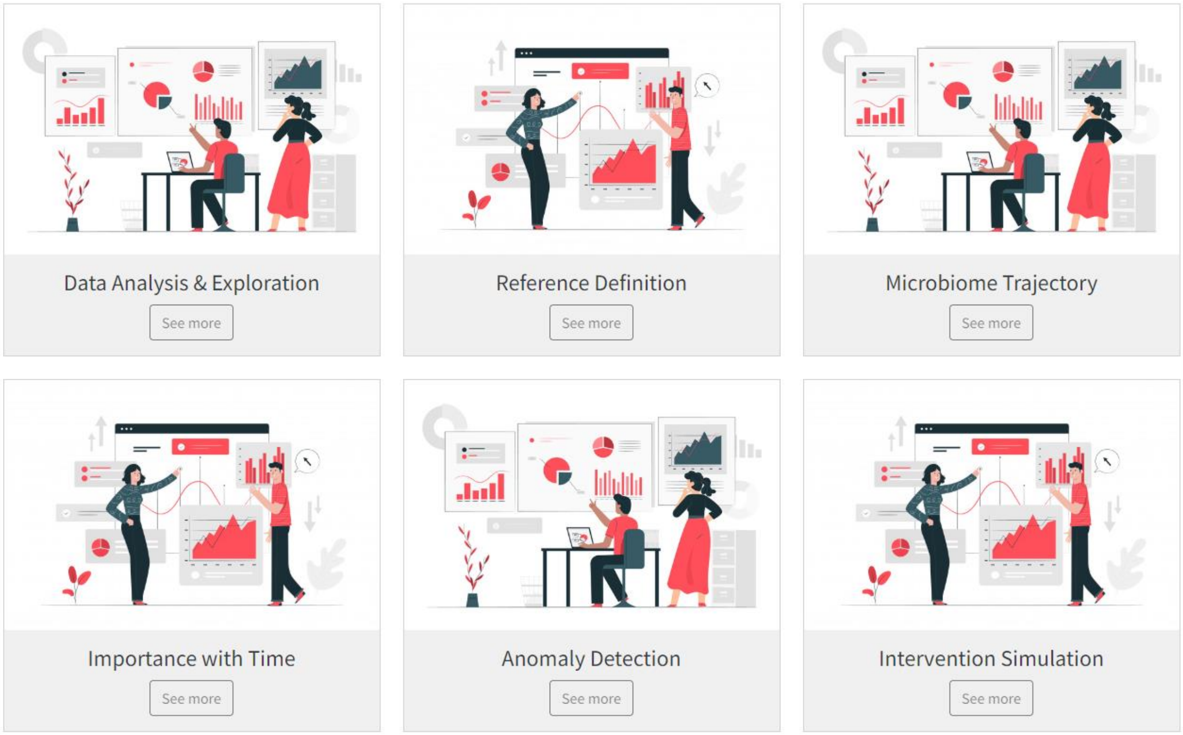

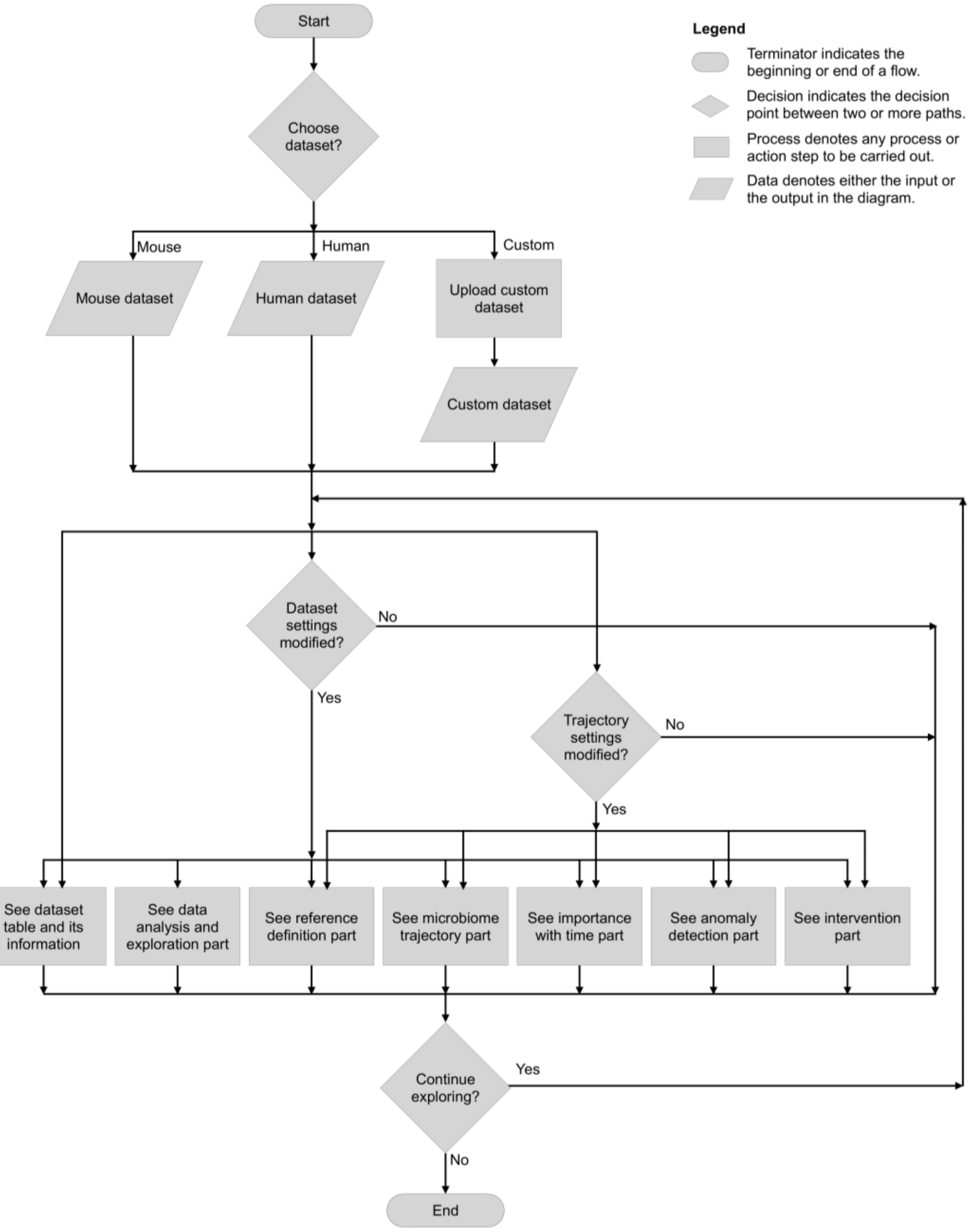

Figure S1: A Flow chart illustrating the different statistical analysis paths a user may take based on the options he chooses, for example, for Dataset Settings or Trajectory settings.

Methods

Supplementary Table 1: methods and algorithms in the microbiome toolbox incorporated from different sources.

Methods	Source	Purpose	Remarks
Machine Learning Algorithms	https://scikit-learn.org/stable/	To learn models to predict Microbiome Maturation Index (MMI)	
SHAP [1]	https://github.com/slundberg/shap	To interpret the predictions of the model using the features it was trained on	
songbird [2]	https://github.com/biocore/songbird	To describe log-fold change of taxa compared to the reference taxa for getting the differential ranking	
CLOUD [3]	Supplementary material of the paper.	Non-parametric detection test for microbiome outliers	Translated and modified for Python
mouseData	https://www.rdocumentation.org/packages/metagenomeSeq/versions/1.14.0/topics/mouseData	To test the methods we used mouse data from RDocumentation	We extract only the metadata and taxa information
splinectomeR [4]	https://github.com/RRShieldsCutler/splinectomeR	Longitudinal statistical analysis tool to compare two non-linear lines	Translated and modified for Python
xgboost	https://github.com/dmlc/xgboost	An optimized distributed gradient boosting library	
scipy	https://www.scipy.org/	Tools for mathematics, science, and engineering	
plotly	https://plotly.com/dash/	Building an interface for the application developed in Python	
catboost	https://github.com/catboost	Gradient boosting on decision trees library	
statsmodels	https://www.statsmodels.org/stable/index.html	To conduct statistical tests and statistical data exploration	

1 Input data

Different types of microbiome data such as compositional and functional data tables, generated from different technologies such as 16s or shotgun metagenomics can be very well used in this toolbox.

2 Microbiome Maturation Index (MMI)

Although the toolbox can be used to analyze any kind of microbiome data that is changing with time or even any other data tables that essentially follow the same longitudinal structure of features changing over time, we have oriented this toolbox more toward an analysis of Early Life Microbiome in infants. Thus, some of the terminologies follow the convention used in the field such as “microbiome maturation” [5].

Briefly, Microbiome Maturation Index (MMI) is defined using the approach from [6]. A similar approach has also been followed by others in the field [7, 8, 9, 10, 11, 12, 13, 14]. Thus, it is a well-used method in the microbiome field particularly for studying Early Life Microbiome progression in infant cohorts.

Here, the actual/chronological age of the subject (infant) is predicted from gut microbiome data (obtained from a fecal sample) using machine learning-based approaches. The term "microbiota age" may encompass both a subject's "microbiota compositional age" and "microbiota functional age". A "microbiota compositional age" may refer to the age which is determined using microbial composition data such as at the genus level or species level. A "microbiota functional age" may refer to the age which is determined using functional data such as pathway modules/submodules or metabolites data. Combined, these can also be termed the "Microbiome Maturation Index" for easier understanding.

3 Statistical methods

The following formulae were used for defining the fit of the trajectory and determining whether a sample is on or off the ELM trajectory.

Least Squares Polynomial Fit

$$\{\widehat{\beta_j}\}_{j=0}^{2} = E \quad where \quad E = \sum_{i=0}^{n} \; |y(\beta_0, \beta_1, \beta_2) - y_i|$$

- n is the number of samples $x_i, y_i, i \in [0, n]$ in the dataset.
- x_i is the independent variable (i.e. age at data collection).
- y_i is the dependent variable to be fitted by model function y with 3 parameters and degree 2 (i.e., MMI).
- $\beta_0, \beta_1, \beta_2$ are parameters of the model to be optimised to minimise E.

Chi-Square: First, consider y_i has uncertainty described by standard deviation σ_i. Second, consider that residuals $r = y(\beta_0, \beta_1, \beta_2) - y_i \sim N$. Then the function:

$$\sum_{i=1}^{n} \; \left[\frac{y_i - y(\beta_1 \dots \beta_m)}{\sigma_i}\right]^2 \sim \chi^2$$

Once we find the best parameters for optimal fit, the probability distribution of χ^2 will be distributed for $n-2$ degrees of freedom. If $y1$ has no uncertainty, $\sigma_i = 1$. This is only used to measure the goodness of a fit. Moreover, we use reduced chi-square χ_r^2 where a good fit should have $\chi_r^2 = 1$.

Confidence Interval: The 95% probability interval around a fit line contains the mean of new values at a specific age of collection value. Iterative resampling of residuals is done 500 times.

$$\left|\hat{\mu}_{y|x0} - \mu_{y|x0}\right| \leq T_{n-2}^{.975}\hat{\sigma}\sqrt{\frac{1}{n} + \frac{\left(x_0 - \underline{x}\right)^2}{\sum_{i=1}^{n} \; \left(x_i - \underline{x}\right)^2}}$$

- $\hat{\mu}_{y|x0} = \sum_{i=0}^{2} \; a_i x_0$ is polynomial fit with a degree of the polynomial equal to 2.
- $\mu_{y|x0}$ is the mean response of new values.
- x_0 is a specific value.
- $T_{n-2}^{.975}$ is the 97.5th percentile of the Student's t-distribution with n-2 degrees of freedom.
- n is the number of samples in the dataset.
- $\hat{\sigma}$ is the standard deviation of the error term in the fit:

$$\hat{\sigma} = \sqrt{\sum_{i=1}^{n} \; \frac{(y_i - \hat{y})^2}{n-2}}$$

Prediction interval: The 95% probability that this interval around a fit line contains a new future observation at specific age at collection value.

$$|\widehat{y_0} - y_0| \leq T_{n-2}^{.975}\hat{\sigma}\sqrt{1 + \frac{1}{n} + \frac{\left(x_0 - \underline{x}\right)^2}{\sum_{i=1}^{n} \left(x_i - \underline{x}\right)^2}}$$

- $\widehat{y_0} = \sum_{i=0}^{2} a_i x_0$ is polynomial fit with a degree of the polynomial equal to 2.
- y_0 is a new observation.
- x_0 is a specific value.
- $T_{n-2}^{.975}$ is 97.5th percentile of the Student's t-distribution with n-2 degrees of freedom.
- n is the number of samples in the dataset.
- $\hat{\sigma}$ is the standard deviation of the error term in the fit:

$$\hat{\sigma} = \sqrt{\sum_{i=1}^{n} \frac{(y_i - \hat{y})^2}{n-2}}$$

4 Comparing Trajectories

We used two different statistical analysis tools that are used to compare the significant difference between the two trajectories:

- Splinectomy longitudinal statistical analysis tools - The methods used are translated from R to Python and accommodated for our project. The original package is called splinectomeR, implemented in R. For more details, please refer to Shields-Cutler RR, et al. Front Microbiol. 2018. Briefly, the test compares whether the area between two polynomial lines is significantly different. Our trajectory lines are the polynomial lines with degrees 2 or 3.
- Linear regression statistical analysis tools: A statistical method based on comparing the two linear lines (line $y = kx + n$). To compare two linear lines, we compare the significant difference in the two coefficients that represent the line k (slope) and n (y-axis intersection).

5 References

[1] Lundberg, S.M., Erion, G., Chen, H. et al. From local explanations to global understanding with explainable AI for trees. Nat Mach Intell 2, 56–67 (2020).

[2] Morton JT, Marotz C, Washburne A, et al. Establishing microbial composition measurement standards with reference frames. Nat Commun. 2019;10(1):2719. Published 2019 Jun 20. doi:10.1038/s41467-019-10656-5

[3] Montassier E, Al-Ghalith GA, Hillmann B, et al. CLOUD: a non-parametric detection test for microbiome outliers. Microbiome. 2018;6(1):137. Published 2018 Aug 6. doi:10.1186/s40168-018-0514-4

[4] Shields-Cutler RR, Al-Ghalith GA, Yassour M, Knights D. SplinectomeR Enables Group Comparisons in Longitudinal Microbiome Studies. Front Microbiol. 2018;9:785. Published 2018 Apr 23. doi:10.3389/fmicb.2018.00785

[5] Dogra SK, Kwong Chung C, Wang D, Sakwinska O, Colombo Mottaz S, Sprenger N. Nurturing the Early Life Gut Microbiome and Immune Maturation for Long Term Health. Microorganisms. 2021;9(10):2110. Published 2021 Oct 7. doi:10.3390/microorganisms9102110

[6] Subramanian S, Huq S, Yatsunenko T, et al. Persistent gut microbiota immaturity in malnourished Bangladeshi children. Nature. 2014;510(7505):417-421. doi:10.1038/nature13421

[7] Wan Y, Zuo T, Xu Z, et al. Underdevelopment of the gut microbiota and bacteria species as non-invasive markers of prediction in children with autism spectrum disorder [published online ahead of print, 2021 Jul 26]. Gut. 2021;gutjnl-2020-324015. doi:10.1136/gutjnl-2020-324015

[8] Depner M, Taft DH, Kirjavainen PV, et al. Maturation of the gut microbiome during the first year of life contributes to the protective farm effect on childhood asthma. Nat Med. 2020;26(11):1766-1775. doi:10.1038/s41591-020-1095-x

[9] Galazzo G, van Best N, Bervoets L, et al. Development of the Microbiota and Associations With Birth Mode, Diet, and Atopic Disorders in a Longitudinal Analysis of Stool Samples, Collected From Infancy Through Early Childhood. Gastroenterology. 2020;158(6):1584-1596. doi:10.1053/j.gastro.2020.01.024

[10] Stewart CJ, Ajami NJ, O'Brien JL, et al. Temporal development of the gut microbiome in early childhood from the TEDDY study. Nature. 2018;562(7728):583-588. doi:10.1038/s41586-018-0617-x

[11] Ho NT, Li F, Lee-Sarwar KA, et al. Meta-analysis of effects of exclusive breastfeeding on infant gut microbiota across populations. Nat Commun. 2018;9(1):4169. Published 2018 Oct 9. doi:10.1038/s41467-018-06473-x

[12] Blanton LV, Charbonneau MR, Salih T, et al. Gut bacteria that prevent growth impairments transmitted by microbiota from malnourished children. Science. 2016;351(6275):10.1126/science.aad3311 aad3311. doi:10.1126/science.aad3311

[13] Bäckhed F, Roswall J, Peng Y, et al. Dynamics and Stabilization of the Human Gut Microbiome during the First Year of Life [published correction appears in Cell Host Microbe. 2015 Jun 10;17(6):852. Jun, Wang [corrected to Wang, Jun]] [published correction appears in Cell Host Microbe. 2015 Jun 10;17(6):852]. Cell Host Microbe. 2015;17(5):690-703. doi:10.1016/j.chom.2015.04.004

[14] Vangay P, Ward T, Gerber JS, Knights D. Antibiotics, pediatric dysbiosis, and disease. Cell Host Microbe. 2015;17(5):553-564. doi:10.1016/j.chom.2015.04.006

Case studies

The Microbiome Toolbox implements methods that can be used for microbiome dataset analysis and microbiome trajectory calculation. The dashboard offers a wide variety of interactive visualizations. Although we believe that python code is the one to be used for detailed analysis, we have still made a web-based tool for (i) quick overview and understanding of the capabilities and functions of this toolbox and (ii) easy use by non-specialized users who do not necessarily use python-code based analysis for their work.

We have dataset checks executed on dataset upload. If the dataset does not follow the formatting rules, the upload error will be reported to the user for suitable modifications of the data format. We provide two demo datasets from studies on mouse and human infants. Both datasets can be downloaded (exported) and can be immediately uploaded for testing as “Custom data”. Thus, these datasets can be used as example references for the users to understand how to format their own data.

Options to change the plots dynamically have been provided now such as change of axis ticks, the number of figures in a panel, height and width of the plot, and many other options as relevant to the figure being displayed. Some of the control over axes, label names, and automatically generated colors are fixed by the framework. For more appearance tuning, we suggest the SVG export option, so the user can further modify the plot in image editing software and save it as a higher resolution.

For easy understanding, users can take demo datasets and look at the analysis section by section, where many useful technical details and references have been provided as relevant for that analytical section. This is illustrated below by two case studies using the mouse and human infants datasets.

Case study 1

We use a mouse study where all mice were fed the same low-fat, plant polysaccharide-rich diet for the first 21 days of the study. At this point 6 of the mice were then switched to a high-fat, high-sugar “Western” diet. We label these groups as “BK” and “Western”. The subsequent changes in the microbial community were then observed over a follow-up of roughly 60 days.

The main thing we examine is the changes in the microbiome in response to dietary differences.

Dimensionality-reduction plots, see Fig 1: In order to visualize samples with lots of feature columns, we use embedding methods like PCA to embed the high-dimensional sample to a lower dimension (2-dim or 3-dim). With this visualization, we hope to catch patterns or clusters that cannot be seen otherwise. If the dataset has a group column, we will be able to distinguish samples based on the group they belong to. By multiple different methods here, we see clear differences in the microbiota profiles of samples distinguished by diet group. From these plots, it also seems that some samples have either been mislabeled or had some other issue, such as didn’t respond to dietary intervention at all, since they cluster with the original diet “BK” group.

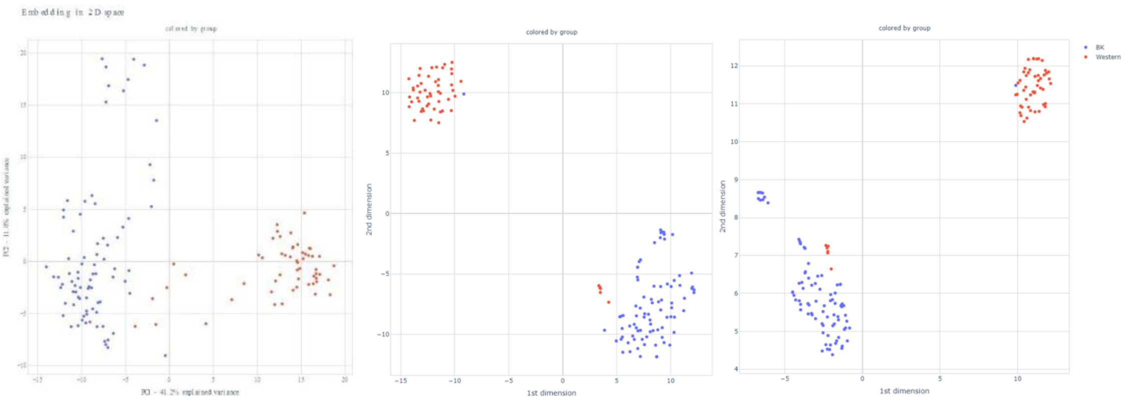

Fig 1. dimensionality reduction methods show clear differences in microbiota patterns by dietary groups (left) PCA (middle) TSNE (right) ISOMAP.

Using the RandomForest-based machine learning model, we can check if we can distinguish the two dietary groups successfully, see Fig 2. The ideal separation between two groups (reference vs. non-reference) will have 100% values detected on the second diagonal. This would mean that the two groups can be easily separated

knowing their taxa abundances and metadata information. Here, an accuracy of 88.54 and an F1-score of 0.79 indicates a very good separation.

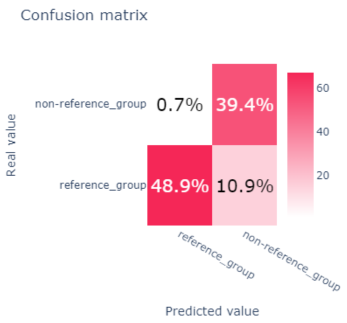

Fig 2. Confusion matrix for the classification task.

Using a SHAP-based analysis, we can identify the microbial signature, i.e., the bacteria taxa driving these differences under two dietary groups, see Fig 3.

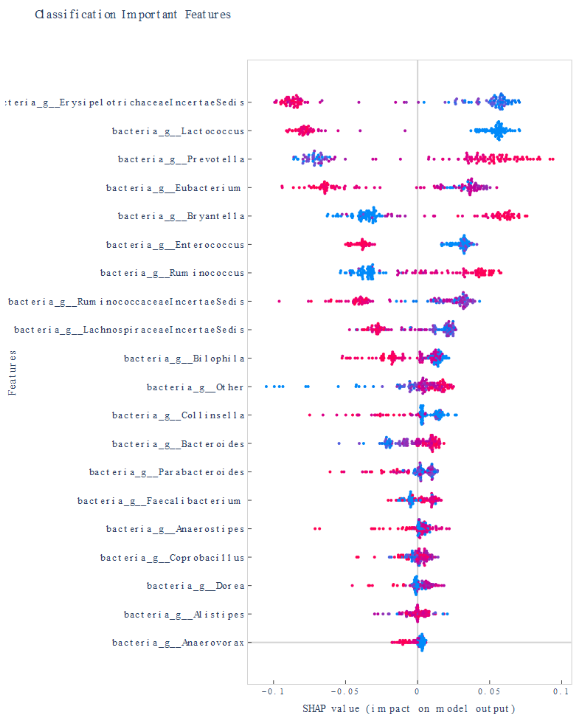

Fig 3. SHAP-based analysis example.

Differences in microbiome changes over time under the influence of external dietary changes here also called succession in ecology can be simply visualized as in Fig 4.

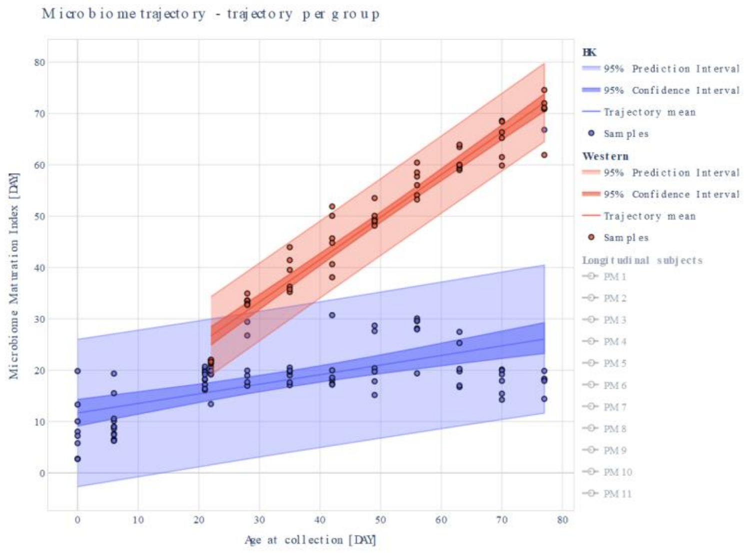

Fig 4. The microbiome trajectories are based on samples from two different types of diets.

Case study 2

Here, we examine the evolution of microbiota of healthy singletons, twins, and triplets from a cohort in Bangladesh. While the study is larger, for the purpose of a demonstration on the dashboard, we take a small snippet of the data for 9 subjects from about 0-6 months of infant age (66 samples).

The main thing we examine is the importance of bacteria building the microbiome trajectory over time as well as identifying the samples diverging from the microbiome trajectory.

Feature extraction plots show what trajectory performance looks like when only working with the top 15 or top 20 bacteria used for the model (when the `TOP_K_IMPORTANT` option is selected). Equivalent thinking goes for the other options (`NEAR_ZERO_VARIANCE` and `CORRELATION`). Metrics used to evaluate the performances of different model sizes are – mean squared error and R-squared. The x-axis shows the number of features used to train the model, the y-axis shows the performance value, see Fig 5.

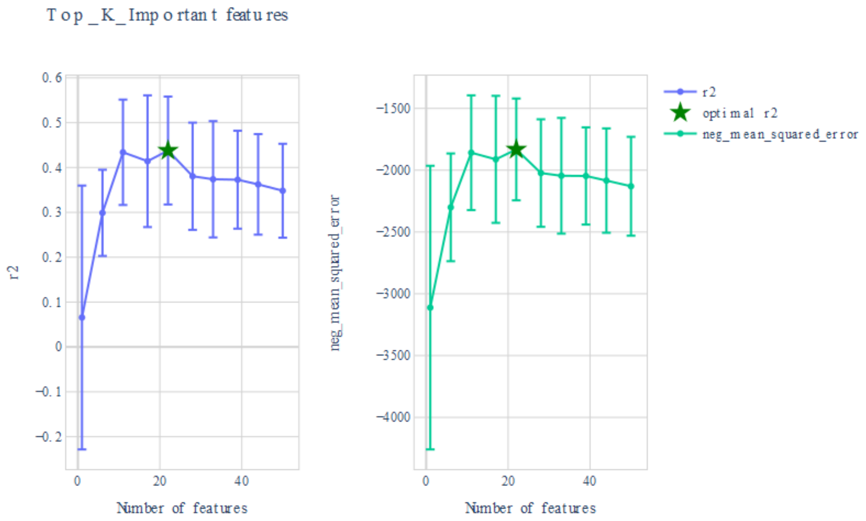

Fig 5. Feature extraction plot showing the optimal number of features that the model can use without a loss in performance.

The importance of the 22 bacteria features used to train the model above can be examined in Fig 6 with the most important bacteria at the top and the least one at the bottom.

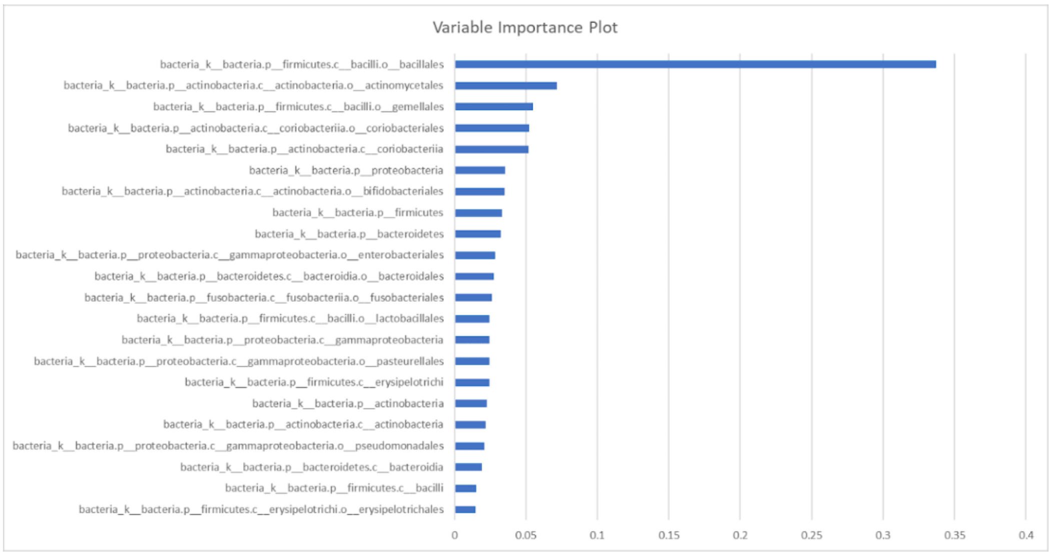

Fig 6. The top important bacteria are sorted by importance on the model result.

Next, the microbiome trajectory is built on the reference samples. Fig 7 below shows the reference samples and confidence intervals with mean. The aim is to capture the relation between x- and y- the best, ideal falling on the diagonal. Here, R-squared obtained is 0.918 with an MAE of 13.266

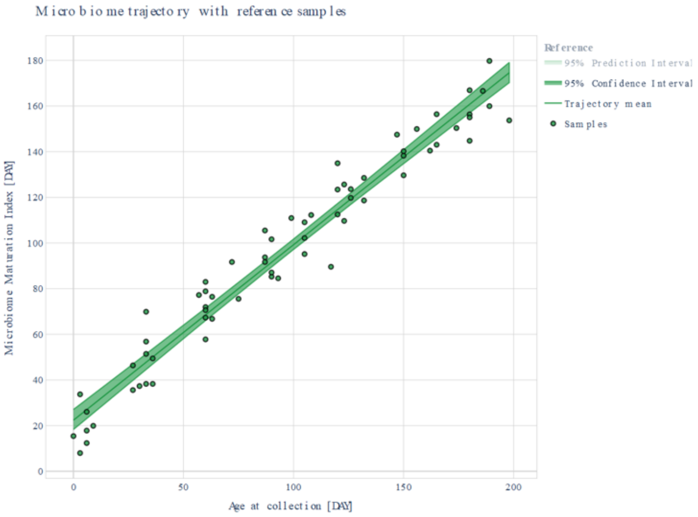

Fig 7. The microbiome trajectory of reference samples.

This dataset can be further split into singletons (18 samples) and twins, triplets (48 samples), and separate trajectories derived for each group. Here, for singletons, we had an R-squared of 0.909, and an MAE of 13.673 days. For, twins and triplets, we had an R-squared of 0.920, and an MAE of 13.114 days

Further, these can be examined separately to test if these groups of infants follow similar trajectories or not, see Fig 8. This can be done both visually as well as by statistical tests. There was no statistically significant difference in trajectories of singletons vs. twins and triplets. The linear p-value (k, n) between Healthy Singletons vs. Healthy Twins Triplets was deemed insignificant as it was >0.05 (0.924, 0.987).

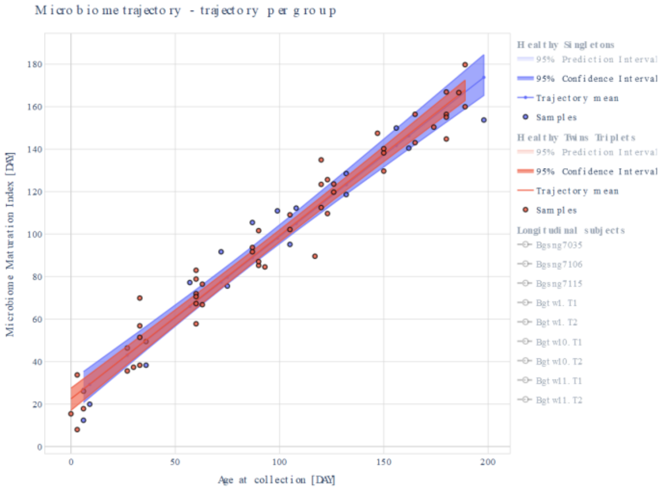

Fig 8. The two microbiome trajectories samples.

We can also longitudinally trace, for example, if twins are following a similar path in and around the reference trajectory or if they are deviant from each other, see Fig 9.

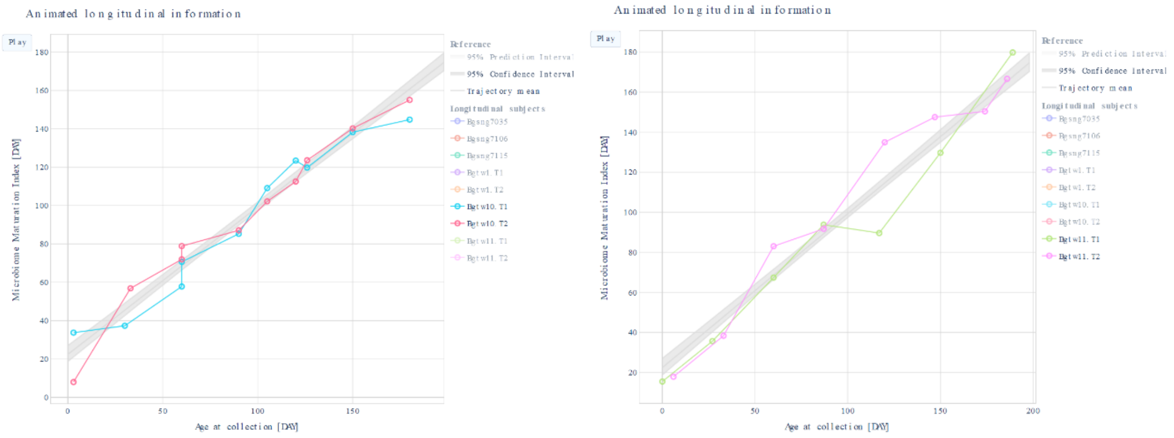

Fig 9. Longitudinal information, samples with similar paths (left), and samples with deviant paths (right).

Further, cross-sectional analyses to examine which are the important bacteria to determine the trajectory as per the time window can be performed, customizable to change the number of top important bacteria and definition of time windows, see Fig 10.

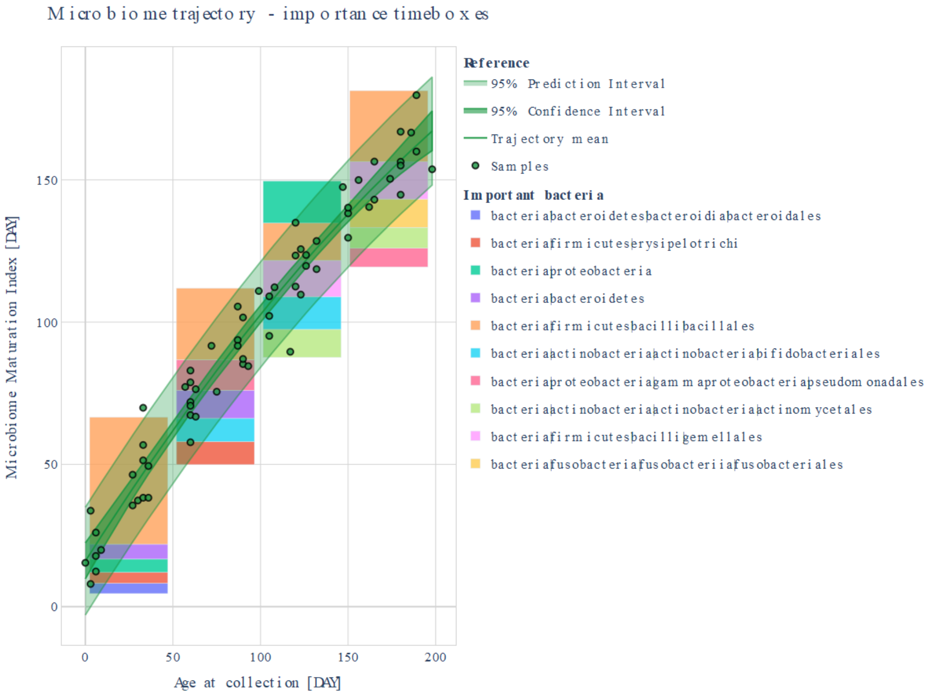

Fig 10. Microbiome trajectory with top important bacteria in each time window.

Outliers can be defined by different statistical measures such as prediction interval, low pass filter, or isolation forest, see Fig 11. Anomalies are detected by examining the samples and identifying which values are out of range making them outliers.

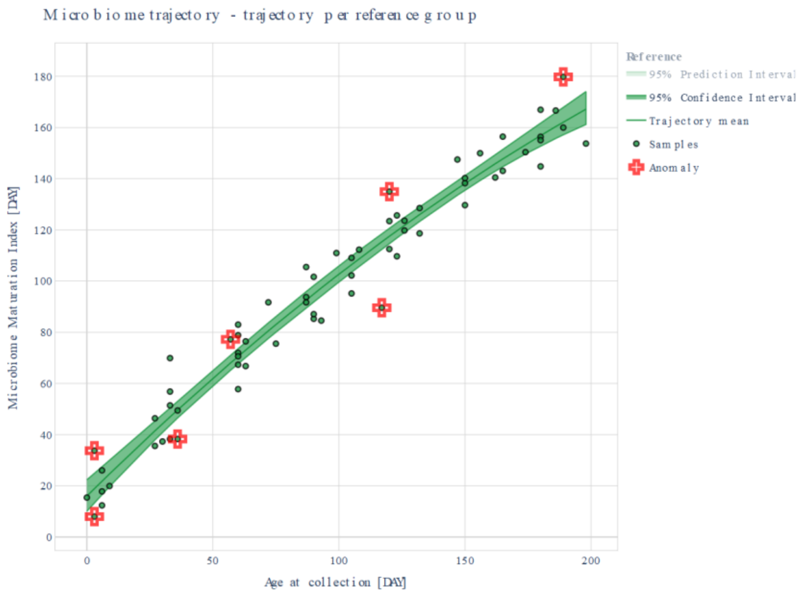

Fig 11. Anomaly detection is where red-colored samples are detected to be anomalies.

Technical Glossary

The Microbiome Toolbox implements methods that can be used for microbiome dataset analysis and microbiome trajectory derivation and related calculations; kindly see the summaries in the two tables below. These are organized by functionality in the Technical Glossary below.

**DATASET**	
**Inputs**	File name<br>Feature columns<br>Normalization<br>Log ratio bacteria (optional)<br>Reference group choice (optional)<br>Time unit
**Processing**	The dataset is read from the **user uploaded csv file** or **available human and mouse datasets**. It consists of several samples collected at different sampling times from multiple subjects. Each sample is represented by several features, particularly bacteria values which specify proportional abundance of different microorganisms, metadata values like diet, country, etc. (optional), sample collection time, sample id, subject id, group is one of the metadata columns renamed to perform group analysis (optional, by default **none**), and reference group (optional, by default all samples are **true**, *i.e.,* belong to the reference).<br>Depending on the **reference group** choice, the definition of reference group samples will be further tuned. Possible choices are **user defined** (default) which leaves the set of reference and non-reference samples unchanged, and **novelty detection** with local outlier factor which searches for the reference samples among non-reference samples (unlabeled samples, that were not set to **true**). The **2 neighbors** and **Bray-Curtis metric** are used as novelty detection parameters. After the novelty detection, the set of reference samples is modified with increased number of reference samples. Note that it only makes sense to use the novelty detection if one specifies set of reference samples and is unsure for the rest.<br>**Differentiation score** is a useful metric value indicating the level of difference between two groups. We are interested in differentiation score between reference and non-reference. Training a binary classifier on features of the samples and reference groups as a label gives us a model whose performance (F1-score) is indicator of two-groups differentiation. The higher the differentiation score, the better the model is in differentiating the two groups.<br>Feature columns define features that the novelty detection algorithm (if selected) will use as explained abve. Supported options are **bacteria columns only**, **metadata columns only**, or **both** (no time variable). Note that this set of feature columns is not necessarily the same as the feature columns used for building the microbiome trajectory.<br>The time unit is mainly used for visualization purposes.<br>If **normalization** is selected, it will normalize each feature column, i.e., each column values will range from 0 to 1. It is a useful preprocessing step in cases where features vary in degrees of magnitude and range.<br>**Log ratio** bacteria is used for calculating the log of the ratio between features and selected bacteria. The log ratio transformation affects the values of bacteria features only and the chosen bacteria, which was used as denominator for log ratio calculations, is removed from feature columns, since after the transformation this column contains only 1 as a value. By default, there is no log ratio bacteria selected (default is **none**) and proportional abundances of different microorganisms are used as provided by the user.
**Outputs**	The plot of bacteria abundances or modified bacteria values if log-ratio transformation is

	performed<br>The heatmap of bacteria abundance or modified bacteria values if log-ratio transformation is performed<br>The plot of ultra-dense longitudinal data with bacteria abundances stacked w.r.t. time<br>Embeddings to 2D/3D space using PCA, UMAP, tSNE<br>Two groups differentiation with confusion matrix after user selects the subset of embedded samples in the low dimensional plot
**Reference**	https://github.com/JelenaBanjac/microbiome-toolbox/blob/main/microbiome/dataset.py

**TRAJECTORY**	
**Inputs**	Dataset (from above)<br>Feature columns<br>Feature extraction<br>Time unit<br>Anomaly type<br>Train indexes
**Processing**	The machine learning algorithm called **RandomForestRegressor** is used to predict sampling times from microbiome composition.<br>Cross-validation resamples data using group k-fold where k is set to **5 folds/splits** and the group is subject id.<br>Grid search with cross-validation is used to find the optimal **number of estimators** for the algorithm which results in the most accurate model predictions. Tested hyperparameter values are **50, 100, and 150**.<br>Feature columns define features that the machine learning algorithm will use for training of the model and inference. Supported options are **bacteria columns only**, **metadata columns only**, or **both** (as provided by the user). The number of feature columns can be further decreased if the user selects one of the feature extraction techniques like **near zero variance**, **correlation**, and **top K important features**. The extraction technique internally runs the same machine-learning algorithm for **10 different thresholds**. The features of the best-performing model are then used as the final smaller feature columns set.<br>Only samples matching the training indices are used for model training (as provided by the user).<br>The time unit is mainly used for visualization purposes.<br>The last part is detecting anomalies using one of the available detection algorithms, **prediction interval**, **low pass filter**, or **isolation forest** which returns indices of anomalous samples. These anomaly indices are later used for visualization purpose only.<br>Comparison of two trajectories is performed between two fitted lines or curves on the MMI data (not actual MMIs but fits), depending on polynomial degree used. Two linear lines (st degree polynomial) are compared using **linear regression statistical analysis** and two curves (N^{th} degree polynomial) are compared using longitudinal statistical analysis method (splinectomeR package).
**Outputs**	The plot of the reference trajectory: one reference trajectory, scattered reference samples<br>The plot of reference groups: two trajectories for both reference and non-reference, scattered samples

	The plot of groups: several microbiome trajectories depending on the groups as provided by the user (e.g., countries, diet-type etc.), scattered group samples<br>The plot of anomalies: reference microbiome trajectory, scattered reference samples, highlighted anomalous samples<br>The plot of time boxes: reference microbiome trajectory, scattered reference samples, timeboxes with specified time block ranges (excluding the anomalous samples) and within time-box feature relative-importance<br>The plot of the intervention simulation: reference microbiome trajectory, timeboxes, and intervention with specified time block ranges (excluding the anomalous samples) and within time-box feature relative-importance<br>The plot of longitudinal sample information: reference microbiome trajectory with a confidence interval, longitudinal samples per subject<br>The plot of feature extraction techniques with 10 different thresholds performance summary (if feature extraction is selected in input)
**Reference**	https://github.com/JelenaBanjac/microbiome-toolbox/blob/main/microbiome/trajectory.py

1 Dataset preparation

Example datasets are given in the dashboard of this Microbiome toolbox

Mouse data

Relevant description of data - All mice were fed the same low-fat, plant polysaccharide-rich diet for the first 21 days of the study. At this point 6 of the mice were then switched to a high-fat, high-sugar "Western" diet. The subsequent changes in the microbial community were then observed over a follow-up of roughly 60 days. Humanized gnotobiotic mouse gut taken from [1, 2]: Twelve germ-free adult male C57BL/6J mice were fed a low-fat, plant polysaccharide-rich diet. Each mouse was gavaged with healthy adult human fecal material. Following the fecal transplant, mice remained on the low-fat, plant polysaccharide-rich diet for four weeks, following which a subset of 6 was switched to a high-fat and high-sugar diet for eight weeks. Fecal samples for each mouse went through PCR amplification of the bacterial 16S rRNA gene V2 region weekly. Details of experimental protocols and further details of the data can be found in [3, 4].

Human data

The dataset for infants from an early life cohort in Bangladesh we used is taken from [5, 6, 7, 8], and only around 66 samples are selected to be used on the web dashboard due to their size.

Custom dataset

For the methods to work, make sure the uploaded dataset has the following columns:

- `sampleID` - a unique dataset identifier, the ID of a sample,
- `subjectID` - an identifier of the subject (i.e., mouse name),
- `age_at_collection` - the time at which the sample was collected, should be in `DAYS`,
- all other required columns in the dataset should be bacteria names which will be automatically prefixed with `bacteria_*` after the upload.

Optional columns:

- `reference_`group - with `True/False` values (e.g. `True` is a healthy sample, `False` is a non-healthy sample); if this column is not specified, it will be automatically created with all `True` values, therefore, all samples will belong to one reference group,
- `group` - the groups that are going to be compared (e.g. country); if this column is not specified, we won't have the visualization of different groups separately,
- `meta_*` - prefix for metadata columns (e.g. c-section becomes meta_csection, etc.),
- `id_*` - prefix for other ID columns (don't prefix `sampleID` nor `subjectID`).

Important: the uploaded dataset should be in a CSV file. In addition, we tested the dashboard with datasets that have less than 100kB. Therefore, if your dataset is bigger, the server might not be able to process it fast enough (there is also a limit of 30 seconds for every request). In this case, we suggest you upload your dataset in a smaller chunk or run the dashboard locally on your computer.

After uploading a Dataset table

Differentiation score: tells us how well the samples from a reference group are separable from the samples from the non-reference group. The measure we use is the F1-score [9] since the underlying model is a binary classifier. Under the hood, we train a binary classifier to differentiate between two groups of samples (reference and non-reference). The binary classification model is `RandomForestClassifier` [10] and we also perform cross-validation with `GroupShuffleSplit` [11]. The parameters we use for the classifier are `n_estimators=140` and `max_samples=0.8`, and they are fixed. To change these values for parameters, you would need to play with the toolbox locally. The result of this classification is the F1 score.

A higher F1 score (closer to 1) means that the samples from the reference group are more likely to be separable from the samples from the non-reference group. Low values of the F1-score (closer to 0) indicate that the samples from the reference group are less likely to be separable from the samples from the non-reference group.

Dataset settings - Feature columns (for novelty detection)

The selection of this option is important only if the user has selected the `NOVELTY_DETECTION` in the Reference group options [12]. Main assumption: the samples that do not belong to the reference group are considered anomalies. We assume that the reference samples are the majority sample representation of the dataset. On the other side, the non-reference samples are the minority and are considered/assumed to be an anomaly of the dataset. Therefore, we use the novelty detection algorithm as an anomaly detection algorithm. More concretely, we use the Local Outlier Factor method (LOF) [13, 14].

The novelty detection algorithm works the following way: take all the samples where `reference_group==True`, find the samples that are the most similar to them, and re-label them to be `True`. The remaining set of samples is labeled `False`, i.e., they are considered anomalies compared to the reference.

Available options are

- `BACTERIA`: the features are only bacteria abundance information,
- `METADATA`: the features are only metadata information,
- `BACTERIA_AND_METADATA`: all features are used for novelty detection,

Important: if your dataset does not have the reference_group column, there is no point in using `NOVELTY_DETECTION`. Select the `USER_DEFINED` option instead.

Dataset settings - Time unit

While it is mandatory to upload a dataset with time format in DAYS, you can specify the format it will be visualized in further data and trajectory analysis.
Available time units: `DAYS, MONTHS, YEARS.`

Dataset settings - Normalization

Data normalization is a very important part of data preparation. It is important to normalize the data to have a mean of 0 and a standard deviation of 1. The goal of normalization is to change the values of numeric columns in the dataset to a common scale, without distorting differences in the ranges of values. For machine learning, every dataset does not require normalization. It is required only when features have different ranges. Therefore, we offer the option to normalize the data.

Dataset settings - Log-ratio bacteria

By default, we use bacteria abundances as the features. However, if you want to use the log-ratio of bacteria abundances w.r.t. the chosen bacteria, select one of the drop-down bacteria. By choosing one of the bacteria, your whole dataset will be transformed to use a log-ratio of bacteria abundances w.r.t. the chosen bacteria. Log ratio is a way to normalize the data.

Dataset settings - Reference group

The reference group is the group of samples that are going to be used as a reference for the microbiome trajectory creation. The reference group is defined in the `reference_group` column with True and False values. The column is not mandatory, but if it is not specified, all samples will automatically be labeled to belong to the reference group (i.e. `reference_group=True`). Information on reference group split analysis can be seen in the Reference Definition card.

There are two options for the reference group:

- `USER_DEFINED`: the user will specify the reference group in the reference_group column,
- `NOVELTY_DETECTION`: the reference group is automatically determined by the novelty detection algorithm.

This can be finetuned further by specifying the feature columns to be used for novelty detection.

Trajectory settings - Feature columns (for the trajectory model)

The feature columns that are used to build a microbiome trajectory can be chosen.

Available options are
- `BACTERIA`: the features are only bacteria abundance information,
- `METADATA`: the features are only metadata information,
- `BACTERIA_AND_METADATA`: all features are used for building the trajectory.

Trajectory settings - Anomaly type

For the anomaly type the default option is to consider anomalies in all the samples that are outside the prediction interval of all the reference samples' trajectory. Information on detected anomalies can be seen in the Anomaly Detection card.

Available options are
- `PREDICTION_INTERVAL`: samples outside the PI are considered to be anomalies,
- `LOW_PASS_FILTER`: the samples passing 2 standard deviations of the mean are considered to be anomalies,
- `ISOLATION_FOREST`: an algorithm that isolates observations by randomly selecting a feature and then randomly selecting a split value between the maximum and minimum values of the selected feature [15, 16, 17].

Trajectory settings - Feature extraction

The feature extraction option specifies additional filtering for columns that are used for microbiome trajectory. Subsequently, information on the performance can be seen in Microbiome Trajectory analyses.

Available options are
- `NONE`: there is no feature extraction, and all feature columns are used,
- `NEAR_ZERO_VARIANCE`: remove features with near zero variance,
- `CORRELATION`: remove correlated features.
- `TOP_K_IMPORTANT`: use only top k important features to build microbiome trajectory.

References:

[1] Joseph Nathaniel Paulson, 2016, metagenomeSeq: Statistical analysis for sparse high-throughput sequencing.
https://bioconductor.org/packages/release/bioc/vignettes/metagenomeSeq/inst/doc/metagenomeSeq.pdf

[2] Package page: metagenomeSeq.
https://bioconductor.org/packages/release/bioc/html/metagenomeSeq.html

[3] Turnbaugh PJ, Ridaura VK, Faith JJ, Rey FE, Knight R, Gordon JI. The effect of diet on the human gut microbiome: a metagenomic analysis in humanized gnotobiotic mice.
https://pubmed.ncbi.nlm.nih.gov/20368178/

[4] Description of the design of human microbiota transplant experiments here.
https://pubmed.ncbi.nlm.nih.gov/20368178/#&gid=article-figures&pid=fig-1-uid-0

[5] Subramanian et al. Persistent Gut Microbiota Immaturity in Malnourished Bangladeshi Children, raw data.
https://gordonlab.wustl.edu/supplemental-data/supplemental-data-portal/subramanian-et-al-2014/

[6] The effects of exclusive breastfeeding on infant gut microbiota: a meta-analysis across populations, some processing included on raw data. https://zenodo.org/record/1304367#.Yrp0EydBw2y

[7] Meta-analysis of effects of exclusive breastfeeding on infant gut microbiota across populations dataset https://zenodo.org/record/1304367#.Yrp0EydBw2y

[8] Ho NT, et al., Meta-analysis of effects of exclusive breastfeeding on infant gut microbiota across populations. https://www.nature.com/articles/s41467-018-06473-x

[9] What is the F1 score? https://deepai.org/machine-learning-glossary-and-terms/f-score

[10] Random forest classifier:

https://scikit-learn.org/stable/modules/generated/sklearn.ensemble.RandomForestClassifier.html

[11] Group shuffle split:

https://scikit-learn.org/stable/modules/generated/sklearn.model_selection.GroupShuffleSplit.html

[12] Novelty and Outlier detection in sklearn.

https://scikit-learn.org/stable/modules/outlier_detection.html#novelty-and-outlier-detection

[13] Local outlier factor LOF.

https://scikit-learn.org/stable/modules/generated/sklearn.neighbors.LocalOutlierFactor.html#sklearn.neighbors.LocalOutlierFactor

[14] Breunig et al. 2000, LOF: identifying density-based local outliers. https://dl.acm.org/doi/10.1145/335191.335388

[15] Isolation forest sklearn. https://scikit-learn.org/stable/modules/generated/sklearn.ensemble.IsolationForest.html

[16] Liu, Fei Tony, Ting, Kai Ming and Zhou, Zhi-Hua. "Isolation forest." Data Mining, 2008. ICDM'08. Eighth IEEE International Conference on.

[17] Liu, Fei Tony, Ting, Kai Ming, and Zhou, Zhi-Hua. "Isolation-based anomaly detection." ACM Transactions on Knowledge Discovery from Data (TKDD) 6.1 (2012)

2 Data analysis and exploration

Some of the methods for data analysis and exploration provided are

- sampling statistics,
- heatmap of taxa abundances w.r.t. time,
- taxa abundance errorbars,
- dense longitudinal data,
- Shannon diversity index and Simpson dominance index (in the GitHub repository of microbiome-toolbox),
- embeddings (different algorithms that we used in 2D and 3D space) with interactive selection and reference analysis.

Taxa Abundances

We plot each bacteria abundance mean and standard deviation w.r.t. time. One plot corresponds to one bacteria with the bacteria name specified in the title. The bacteria can be turned on/off by clicking on its corresponding label in the legend.

Taxa Abundances Heatmap

Another way to visualize taxa abundances is by using the heatmap. All taxa are visualized on one plot: the y-axis shows the bacteria name and the x-axis shows the time point. Color intensity indicates the abundance value.

Dense Longitudinal Data

One subplot corresponds to the longitudinal bacteria data of one subject. Bacteria abundances are stacked on top of each other. One color corresponds to one bacteria. If there are more bacteria than colors in a color palette (tab20), you can specify another color palette, see available options here: https://matplotlib.org/stable/tutorials/colors/colormaps.html

Embedding in 2D space

In order to visualize samples with lots of feature columns (columns used to build a microbiome trajectory), we use embedding methods such as PCA, TSNE, ISOMAP, and UMAP to embed the high-dimensional sample to a lower dimension (2-dim or 3-dim). With this visualization, we hope to catch patterns or clusters that cannot be seen otherwise. If the dataset has a `group` column, we will be able to distinguish samples based on the group they belong to.

Embedding in 2D space - Interactive Analysis

Interactive version of embedding samples to a low-dimensional space. Here we have support for 2D sample selection. After the selection, we build a binary classifier that tries to differentiate between selected and non-selected samples on a 2D plot. The confusion matrix and F1 score are reported as indicators of this two-groups differentiation. To use an interactive option: click on the plotting toolbox the Lasso Select option, select the samples you want to group, and wait for the explanatory information to load (with a confusion matrix).

3 Reference definition

There are two ways to define the reference set in the dataset:

- `USER_DEFINED` (i.e. predefined by the user): all samples that belong to the reference are specified by the user in the uploaded dataset (samples where `reference_group==True`). Other samples are considered to be non-reference samples. If the uploaded dataset does not have a `reference_group` column, it will be created automatically with all `True` values. This means that all samples will be considered reference samples.

- `NOVELTY_DETECTION` (i.e. unsupervised anomaly detection) performs novelty and outlier detection. The algorithm uses the user's reference defined as a start (samples where `reference_group==True`) and decides whether a new observation from unlabeled samples belongs to the reference or not. We use the LocalOutlierFactor [1] method with the default Bray-Curtis [2] distance metric and 2 neighbors. These parameters can be modified on the Home page. The features used for this model are specified under the Dataset Settings feature columns option on the Home page (when the novelty detection option is selected). These features are not necessarily matching the feature columns used for building the microbiome trajectory. If the dashboard user selects the novelty detection option, it might not yield the optimal results for default parameters (fixed parameters cannot be generalized across different datasets). Hence, we suggest playing with the parameters (metric and number of neighbors).

We also analyze the features important in each of the groups. To find the features that differentiate the two groups (reference vs. non-reference group), we train the binary classification model `RandomForestClassifier` [3] and perform cross-validation with `GroupShuffleSplit` [4]. The parameters we use for the classifier are `n_estimators=140` and `max_samples=0.8`, and they are fixed. To change these values for parameters, you would need to play with the toolbox locally. The confusion matrix enables insight into how good the separation between the two groups is.

References:

[1] Local outlier factor (LOF):

https://scikit-learn.org/stable/auto_examples/neighbors/plot_lof_outlier_detection.html

[2] Bray-Curtis dissimilarity: https://en.wikipedia.org/wiki/Bray%E2%80%93Curtis_dissimilarity

[3] Random forest classifier:

https://scikit-learn.org/stable/modules/generated/sklearn.ensemble.RandomForestClassifier.html

[4] Group shuffle split:

https://scikit-learn.org/stable/modules/generated/sklearn.model_selection.GroupShuffleSplit.html

4 Microbiome trajectory

The microbiome trajectory is often used in microbiome research as a visualization showing the microbiome development with time. The reference samples are the samples that are used to build the microbiome trajectory. Using machine learning algorithms, we predict a Microbiome Maturation Index (MMI) primarily as a function of the microbiome composition or similar (eg., metabolites or CAZymes composition). A smooth fit is then used to obtain the trajectory. With the visualizations below we hope to discover the microbiome trajectory of a given dataset.

Available techniques to decrease the size of the model while still keeping its performance are:
- `TOP_K_IMPORTANT`: features selection based on the smallest mean absolute error,
- `NEAR_ZERO_VARIANCE`: remove near zero variance features,
- `CORRELATION`: remove correlated features.

To ensure the performance is still good, we show the plots in the Feature extraction part.

Microbiome trajectory plots contain the following information:
- only mean line,
- only line with prediction interval and confidence interval,
- line with samples,
- longitudinal data, every subject,
- coloring per group (e.g., per country).

Measuring the trajectory performance (all before plateau area):
- mean absolute error (MAE) error between the predicted value (MMI, predicted sampling time) and actual value (actual sampling time) (goal: smaller),
- R^2, R-squared score (goal: bigger), percent of variance captured,
- Pearson correlation (MMI, age_at_collection),
- Prediction Interval (PI) is a prediction interval of 95%, the interval in which we expect the healthy reference to fall in (goal: smaller),
- The standard deviation of the error,
- Visual check.

We used two different statistical analysis tools that are used to compare the significant difference between the two trajectories:

- Splinectomy longitudinal statistical analysis tools: The method used is translated from R to Python and accommodated for our project. The original package is called `splinectomeR` [1], implemented in R. For more details please check [2]. In short, the test compares whether the area between two polynomial lines is significantly different. Our trajectory lines are the polynomial lines with degrees 2 or 3.

- Linear regression statistical analysis tools: A statistical method based on comparing the two linear lines (line y=k*x+n). To compare two linear lines, we compare the significant difference in the two coefficients that represent the line k (slope) and n (y-axis intersection).

<u>Reminder</u>: a p-value less than 0.05 (typically ≤ 0.05) is statistically significant (i.e. lines are different). A p-value higher than 0.05 (> 0.05) is not statistically significant and indicates strong evidence for the null hypothesis (H0: two lines have similar slope/intersection/etc.). This means we retain the null hypothesis and reject the alternative hypothesis.

<u>Reference trajectory</u>: The microbiome trajectory is built on the reference samples. The plot shows reference samples, their prediction, and confidence intervals with mean.

<u>Reference groups</u>: If the dataset has reference and non-reference samples, both lines will be visualized separately with their corresponding samples, prediction, and confidence intervals with mean.

Groups: If the dataset has several groups, all lines will be visualized separately with their corresponding samples, prediction, and confidence intervals with mean.

Longitudinal information: Shows animated longitudinal information of reference samples.

References:

[1] R package: https://github.com/RRShieldsCutler/splinectomeR

[2] Shields-Cutler et al. SplinectomeR Enables Group Comparisons in Longitudinal Microbiome Studies: https://www.frontiersin.org/articles/10.3389/fmicb.2018.00785/full

5 Importance with time

This type of analysis is useful if we are interested to see which bacteria are important in what time block for a microbiome trajectory that is built on the reference samples.

Importance of different bacteria and their abundances across time blocks:
- bacteria importance is stacked vertically where the size of each bacteria sub-block represents its importance in that time block,
- values shown on mouse hover the box represents the mean and standard deviation of its abundance in that time block (Note: we tested mean, geometric mean, robust mean, and median represented data the best for our data. In this toolbox, we have support for any average function a user may want),
- the total height of the box is fixed in all-time blocks,
- can choose a few important bacteria for a time interval.

6 Anomaly detection

There is support for detecting anomalies in three different ways:

- `PREDICTION_INTERVAL`: samples outside the Prediction Interval (PI) are considered to be anomalies; the fixed parameter for the algorithm is the degree of the polynomial line, where `degree = 3` (i.e. the microbiome trajectory approximation line is non-linear),

- `LOW_PASS_FILTER`: the samples passing 2 standard deviations of the mean are considered to be anomalies; fixed parameters for the algorithm are `window = 10` (window size for the filter, see [4]) and `number_of_std = 2` (i.e. samples that are outside 2 standard deviations of the mean are considered to be anomalies),

- `ISOLATION_FOREST`: unsupervised anomaly detection algorithm on longitudinal data to obtain what samples are anomalous [1, 2, 3]. The algorithm isolates observations by randomly selecting a feature and then randomly selecting a split value between the maximum and minimum values of the selected feature; fixed parameters for the algorithm are `window = 5` (used to calculate moving average) and `outlier_fraction = 0.1` (i.e. we expect to have around 10% of anomalies in the dataset).

By default, the anomaly is a sample that is outside the prediction interval of the microbiome trajectory that is built on reference samples. Note: currently there is no option to modify fixed parameters of the anomaly algorithms within the dashboard. To modify these parameters, please use the toolbox locally.

References:

[1] Isolation forest (IF) sklearn

https://scikit-learn.org/stable/modules/generated/sklearn.ensemble.IsolationForest.html

[2] Liu, Fei Tony, Ting, Kai Ming, and Zhou, Zhi-Hua. “Isolation forest.” Data Mining, 2008. ICDM’08. Eighth IEEE International Conference,

[3] Liu, Fei Tony, Ting, Kai Ming and Zhou, Zhi-Hua. “Isolation-based anomaly detection.” ACM Transactions on Knowledge Discovery from Data (TKDD) 6.1 (2012).

[4] Deciding on window size https://www.google.com/search?q=filter+window+size&oq=filter+window+size

7 Intervention simulation

The intervention simulation is a technique we propose to use in order to return an anomaly back to the reference trajectory. For the anomaly, this is done by modifying the values of those bacteria that were found to be top important for reference samples. In other words, the values of these bacteria for the anomaly are substituted by the mean values from the reference samples.

The intervention simulation consists of suggesting the bacteria values to change (or log-ratio values to change) in order to bring back the sample to the reference microbiome trajectory.

If an anomaly is not back on the reference trajectory, some of the possible reasons are:

- the time block in which the anomaly is located is not wide or small enough,
- the reference samples have samples that should not be considered as reference samples,
- the number of top important bacteria to consider is not sufficient to help an anomaly to become a reference sample.
